# STEMDiff: A Wavelet‐Enhanced Diffusion Model for Physics‐Informed STEM Image Generation

**DOI:** 10.1002/advs.202508266

**Published:** 2025-09-27

**Authors:** Yihui Bao, Xinyi Lu, Yanyan Xia, Zhencheng Ye, Houyang Chen

**Affiliations:** ^1^ School of Information Science and Engineering East China University of Science and Technology Shanghai 200237 P. R. China; ^2^ Chongqing Institute of Green and Intelligent Technology Chinese Academy of Sciences Chongqing 400714 P. R. China; ^3^ Chongqing School University of Chinese Academy of Sciences Chongqing 400714 P. R. China

**Keywords:** diffusion model, materials design, STEM image generation, STEM, STEMDiff

## Abstract

Machine learning has emerged as a powerful tool for analyzing scanning transmission electron microscopy (STEM) images, yet its widespread application remains constrained by the scarcity of annotated training data. While deep generative models offer a promising solution, they typically struggle to reproduce the complex high‐frequency components that define experimental STEM images. Here, STEMDiff, a conditional diffusion model that transforms simple binary labels derived from crystal structures into realistic STEM images through a physical information embedding strategy, is proposed. By developing a novel Discrete Wavelet Transform‐based skip‐connection architecture, the high‐frequency bias inherent in diffusion models are addressed, enabling the preservation of experimental noise characteristics. This approach generates images that are quantitatively nearly indistinguishable from experimental data (17 fold improvement over previous methods) while retaining ground truth structural information. Fully convolutional networks trained exclusively on these synthetic images achieve high‐precision atomic column detection in experimental STEM images of WSe_2_ and graphene, despite the presence of substantial background noise and contamination. This approach effectively eliminates the need for laborious manual annotation, providing a scalable solution to the data bottleneck in STEM image analysis. The principles underlying STEMDiff can extend to other scientific imaging modalities, accelerating advancements in materials design for water treatment.

## Graphical abstract

STEMDiff, which is a conditional diffusion model that generates realistic STEM images from crystal structure‐derived binary labels, is proposed, overcoming high‐frequency bias via a Discrete Wavelet Transform‐based architecture. It produces experimentally indistinguishable images, enabling annotation‐free training of networks for precise atomic detection in noisy data. This scalable tool accelerates materials discovery in energy storage, catalysis, and water treatment.

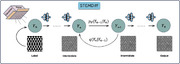

## 1 Introduction

Advancing the understanding of materials at the atomic scale is fundamental to designing next‐generation functional materials for applications ranging from quantum computing to heterogeneous catalysis and energy storage, as well as water treatment.^[^ ^1^, ^2^, ^3^, ^4^, ^5^ ^]^ Scanning transmission electron microscopy (STEM) has emerged as an indispensable tool in this pursuit, enabling direct visualization of atomic arrangements with sub‐angstrom resolution.^[^ ^6^, ^7^ ^]^ However, the extraction of comprehensive quantitative information from these complex images, such as encompassing atomic positions,^[^ ^8^, ^9^ ^]^ defects,^[^ ^10^, ^11^ ^]^ strain fields,^[^ ^12^, ^13^ ^]^ and compositional variations,^[^ ^14^ ^]^ increasingly requires sophisticated machine learning (ML) approaches to handle the scale and complexity of modern electron microscopy datasets.

The ability to precisely characterize nanoscale material features has profound implications for chemical process development and optimization. In heterogeneous catalysis, atomic‐level understanding of active site distributions, particle morphologies, and support interactions directly influences reaction pathways, selectivity, and catalyst lifetime. These structure‐property relationships critically impact process design parameters, including reactor configurations, operating conditions, and economic viability of chemical processes.

A critical bottleneck in deploying ML for STEM image analysis is the scarcity of high‐quality annotated training data. The acquisition of experimental STEM images is time‐intensive and limited by access to specialized instruments, while manual annotation introduces human bias and severely restricts dataset size. Conventional approaches that rely on physics‐based simulations followed by manual addition of experimental artifacts are not only labor‐intensive but also fall short in capturing the statistical complexity of real experimental images. These artifacts include detector noise,^[^ ^15^ ^]^ drift‐induced distortions,^[^ ^16^ ^]^ probe instabilities,^[^ ^17^ ^]^ and sample contamination,^[^ ^18^ ^]^ all of which significantly influence image characteristics yet are challenging to model analytically.

Recent attempts to bridge this simulation‐experiment gap have explored generative adversarial networks (GANs),^[^ ^19^ ^]^ which have shown promise in creating realistic STEM images. For instance, Khan et al.^[^ ^20^ ^]^ demonstrated that cycle‐consistent GANs can transform simulated STEM images into experimental‐like ones with a Fréchet Inception Distance (FID) score of 0.35. However, GANs often suffer from training instability, mode collapse, and limited interpretability, constraining their practical utility for scientific imaging applications where physical accuracy is paramount.

Diffusion models^[^ ^21^, ^22^, ^23^ ^]^ represent a fundamentally different approach to generative modeling, learning the underlying noise distribution of data through a progressive denoising process. Unlike GANs, these models offer improved training stability, better mode coverage, and a more principled probabilistic framework. Despite these advantages, the commonly used U‐Net architectures,^[^ ^24^ ^]^ which work as a denoising module in diffusion models, exhibit an inherent high‐frequency bias,^[^ ^25^, ^26^ ^]^ i.e., they tend to prioritize low‐frequency image components while inadequately capturing the high‐frequency details that are critical in experimental STEM images. This limitation is particularly problematic for scientific applications where fine details often contain essential physical information.

Here, we propose STEMDiff, a conditional diffusion model trained on physically simulated STEM images that transforms simple binary label maps derived directly from crystal structures into highly realistic STEM images through a physics‐informed approach. The key innovation lies in the development of Discrete Wavelet Transform (DWT)‐based Noise Retaining Blocks that are strategically integrated into the model architecture. These specialized blocks effectively isolate and preserve the high‐frequency components characteristic of experimental STEM images, overcoming the high‐frequency bias inherent in standard diffusion models. This wavelet‐based approach proves particularly advantageous for electron microscopy images, where atomic‐level details and experimental artifacts occupy specific frequency bands that must be accurately reproduced. The performance of STEMDiff surpasses previous approaches by a substantial margin, achieving an FID score of 0.02, representing a 17 fold improvement over state‐of‐the‐art GAN‐based methods. Quantitative analysis using Kullback‐Leibler (KL) divergence further confirms that the statistical distribution of STEMDiff‐generated images closely aligns with that of experimental data. More importantly, these synthetic images retain the physical fidelity required for downstream analytical tasks, as demonstrated by the successful training of atomic column detection networks using exclusively STEMDiff‐generated data.

STEMDiff provides a practical approach for physics‐informed synthetic data generation that could transform how ML is applied across scientific imaging modalities. By enabling the creation of virtually unlimited training data with ground truth annotations, this approach addresses a fundamental challenge in scientific machine learning, i.e., bridging the gap between simulation and experiment while preserving physical accuracy. As the volume and complexity of electron microscopy data continue to grow, such tools will become increasingly essential for extracting meaningful insights and accelerating materials discovery. Beyond electron microscopy, the physics‐informed generative framework of STEMDiff offers a blueprint for synthetic data generation in other modalities, such as X‐ray tomography or optical imaging, where data scarcity hinders artificial intelligence (AI) driven discovery. This positions our work at the interdisciplinarity of materials science, computational chemistry, and ML, with potential to impact fields like water treatment, renewable energy, and nanotechnology.

## 2 Methodology

### 2.1 Architectural Design and Theoretical Framework of STEMDiff

The fundamental challenge in STEM image generation lies in creating realistic simulations that capture both structural accuracy and experimental artifacts. While conventional simulation approaches often struggle with this dual requirement, we address it through a novel generative framework combining diffusion models with wavelet theory, denoted as STEMDiff modeling.

**Figure 1:**
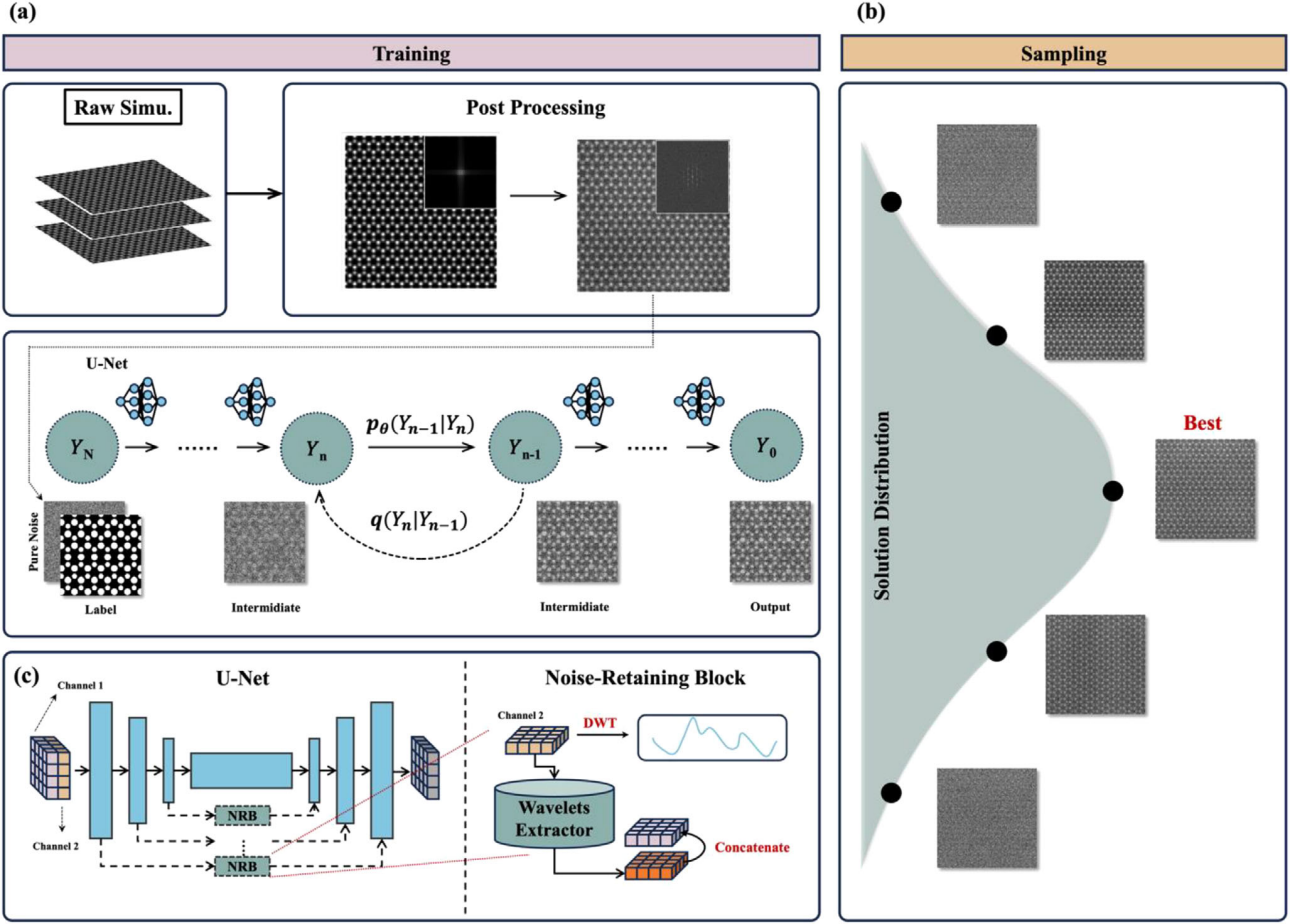
Schematic of the major components in STEMDiff. a) The training process of the proposed “Label to STEM” conditional diffusion model. At step n, the modified U‐Net neural network takes a simulated STEM image ***I***_***stem***_ and a noisy image ***J***_***n***_ as inputs. Each simulated image is paired with a binary label map derived by projecting the atomic coordinates of the input crystal structure onto a 2D grid, where atomic column regions are labeled as 1 and background regions as 0. b) The sampling process of the proposed “Label to STEM” conditional diffusion model, c) The network architecture incorporates a customized skip connection mechanism employing the Noise‐Retaining Block (NRB) to ensure experimental noise signatures remain intact throughout the STEM image generation pipeline.

STEMDiff is a high‐precision image generation framework built upon conditional diffusion models, tailored to enable structure‐preserving synthesis of Scanning Transmission Electron Microscopy (STEM) images that resemble experimental data. Its fundamental architecture integrates three core components: 1) forward diffusion process: a progressive noise‐injection pathway that systematically transforms images into noise, 2) reverse denoising process: a neural denoising process that reconstructs authentic images from noise, and 3) conditional guidance mechanism: a conditional steering mechanism guided by binary atomic positions. Unlike previous approaches utilizing GANs^[^ ^19^ ^]^ or explicit texture representations (e.g., Gram matrix^[^ ^27^ ^]^), STEMDiff leverages the robust mathematical foundations of diffusion probabilistic theory, with the Denoising Diffusion Probabilistic Model (DDPM)^[^ ^28^ ^]^ serving as the backbone. This framework (**Figure** **1**) provides significantly improved training stability and better preservation of physical accuracy in the generated images. This design not only enhances fidelity but also enables scalable dataset generation for training ML models in resource‐limited settings, such as industrial materials research.

It is important to emphasize that although STEMDiff generates images during inference using binary structural labels and Gaussian noise as inputs, the model is trained on paired datasets comprising precisely simulated STEM images and their associated label maps. These simulated images are derived from ab initio physical simulations, providing an essential basis for the ability of STEMDiff to generate images from structural information.

In this study, a binary label map denotes a 2D matrix matching the resolution of the simulated STEM image. It is created by projecting atomic positions from the input crystal structure file onto a 2D grid, where pixels representing atomic columns receive a value of 1, and background areas are assigned 0. As such, this binary map acts as a streamlined depiction of the structure, maintaining the spatial arrangements and rough outlines of atomic columns. The label maps are produced using a custom Python script that ensures perfect alignment with the simulated STEM images generated from identical atomic coordinates.

#### 2.1.1 Forward Diffusion Process

The forward noise‐injection process establishes a probabilistic Markov chain that methodically transforms an original simulated STEM image y₀ into pure noise through N timesteps:

(1) $$\begin{array}{c} q\;\left(y_{1:N}|y_{0}\right)=\prod_{n=1}^{N}q\left(y_{n}|y_{n-1}\right)\; \end{array}$$

Each transition in the chain follows a precisely calibrated Gaussian distribution with systematically increasing variance:

(2) $$\begin{array}{c} q\;\left(y_{n}|y_{n-1}\right)=N\left(y_{n}|\sqrt{\alpha_{n}}y_{n-1},\left(1-\alpha_{n}\right)I\right) \end{array}$$

where α_n_ ∈ (0,1) represents variance scaling factors that regulate the noise intensity at each step, with the coefficient $\sqrt{\alpha_{n}}$ ensuring numerical stability as the sequence progresses. Through analytical marginalization, we can express the noise distribution at any timestep n as:

(3) $$\begin{array}{c} q\;\left(y_{n}|y_{0}\right)=N\left(y_{n}|\sqrt{{\bar{\alpha }}_{n}}y_{0},\left(1-{\bar{\alpha }}_{n}\right)I\right) \end{array}$$

where ${\bar{\alpha }}_{n}=\prod_{s=1}^{n}\alpha_{s}$ defines the cumulative noise scaling factor.

#### 2.1.2 Reverse Denoising Process

The core of STEMDiff lies in its denoising reconstruction pathway, transforming from standard Gaussian noise y_N_ back into coherent STEM imagery guided by a structural binary label map x:

(4) $$\begin{array}{c} p_{\theta }\;\left(y_{0:N}|x\right)=p\left(y_{n}\right)\prod_{t=1}^{N}p_{\theta }\left(y_{t-1}|y_{t},x\right) \end{array}$$

Each reconstruction step operates as a conditional Gaussian transformation:

(5) $$\begin{array}{c} p_{\theta }\;\left(y_{n-1}|y_{n},x\right)=N\left(y_{n-1}|\;\mu_{\theta }\left(x,y_{n},\alpha_{n}\right),\sigma_{n}^{2}I\right) \end{array}$$

Our optimization objective focuses on precise noise prediction, training the neural network *g*_ϕ_ to accurately estimate the noise component introduced during the forward process, with the loss function:

(6) $$\begin{array}{c} L=E\left(x,y\right)E_{\varepsilon ∼N\left(0,1\right),n}∥g_{\phi }\left(x,y_{n},\alpha_{n}\right)-\varepsilon ∥ \end{array}$$

#### 2.1.3 High‐Frequency Preservation through Noise Retaining Blocks (NRB)

A critical limitation we identified in conventional diffusion architectures when applied to scientific imaging is their inherent preference for reconstructing low‐frequency information at the expense of crucial high‐frequency details. The frequency bias becomes particularly problematic for STEM imaging, where atomic‐scale features and experimental artifacts predominantly occupy high‐frequency spectral ranges.

To overcome this fundamental limitation, we introduce Noise Retaining Blocks (NRBs) based on multi‐resolution wavelet analysis. These specialized architectural components implement a structured four‐phase process (Figure 1c):

1. Multi‐resolution decomposition: The feature representation x₀∈ℝ^H×W×2^ undergoes discrete wavelet transformation into four complementary frequency sub‐representations: x_ll_, x_lh_, x_hl_, x_hh_, capturing approximation and directional detail coefficients.
2. High‐frequency component isolation: The x_hh_ sub‐representation, containing diagonal high‐frequency information crucial for experimental authenticity, is selectively preserved at its reduced spatial dimensions ℝ^H/2×W/2^.
3. Inverse wavelet transformation: The isolated high‐frequency components undergo inverse wavelet transformation, reconstructing these critical elements while preserving their essential characteristics.
4. Adaptive integration: The reconstructed high‐frequency elements are systematically integrated into subsequent processing layers through modified skip connections, fundamentally altering the information propagation compared to conventional U‐Net architectures.^[^ ^29^ ^]^

This wavelet‐based approach transforms how frequency information flows through the network, enabling STEMDiff to preserve and reconstruct the intricate high‐frequency details that characterize authentic experimental STEM observations. By replacing conventional skip connections with NRBs throughout the architecture, we achieve substantially improved fidelity while maintaining computational efficiency.

### 2.2 Raw Simulated Image Enhancement Protocol

The development of STEMDiff necessitated the implementation of sophisticated pre‐processing methodologies to bridge the realism gap between simulations and experimental reality. Our comprehensive enhancement protocol consists of the following sequential steps:

1. Statistical noise injection: Introduction of physically motivated Poisson noise patterns at the pixel level, accurately replicating the quantum statistical fluctuations inherent in experimental electron detection systems.
2. Geometric distortion modeling: Application of a sequence of spatial geometric manipulations, including horizontal shear, vertical compression, and jittering.
3. Environmental noise simulation: Integration of calibrated Gaussian noise distributions to mimic unpredictable variations encountered in real‐world imaging environments.
4. Contamination effect reproduction: Extraction and incorporation of low‐frequency background components from experimental STEM images using Fourier filtering to replicate localized ultra‐bright regions caused by sample contamination.

This comprehensive enhancement strategy, combined with the wavelet‐enhanced diffusion architecture, enables STEMDiff to generate synthetic STEM images with unprecedented experimental fidelity while preserving ground truth structural information critical for ML applications in materials science. The implementation and training details are provided in the supporting information.

## 3 Results and Discussions

### 3.1 Generation of Experimentally Authentic STEM Images via STEMDiff

STEMDiff demonstrates unprecedented capability in bridging the persistent gap between computational simulations and experimental reality in STEM. To systematically evaluate this capability, by taking monolayer tungsten diselenide (WSe_2_) as an example, we conducted comparative analyses between three distinct image classes, i.e., raw simulated images, STEMDiff‐generated outputs, and experimental STEM images, examining both their spatial and frequency‐domain characteristics.

Initially, we established a baseline by generating idealized STEM simulations using the abTEM^[^ ^30^ ^]^ and atomic simulation environment (ASE) Python packages,^[^ ^31^ ^]^ incorporating precise electron optical parameters matched to experimental conditions. However, these conventional simulated images intrinsically lack critical experimental features, including detector noise,^[^ ^15^ ^]^ drift distortions,^[^ ^16^ ^]^ probe jittering,^[^ ^17^ ^]^ lens aberrations,^[^ ^32^ ^]^ thickness variations,^[^ ^9^ ^]^ and surface contamination phenomena.^[^ ^18^ ^]^ These experimental realities collectively contribute to the characteristic signature of authentic STEM observations that conventional simulation methodologies fundamentally cannot reproduce.

**Figure 2:**
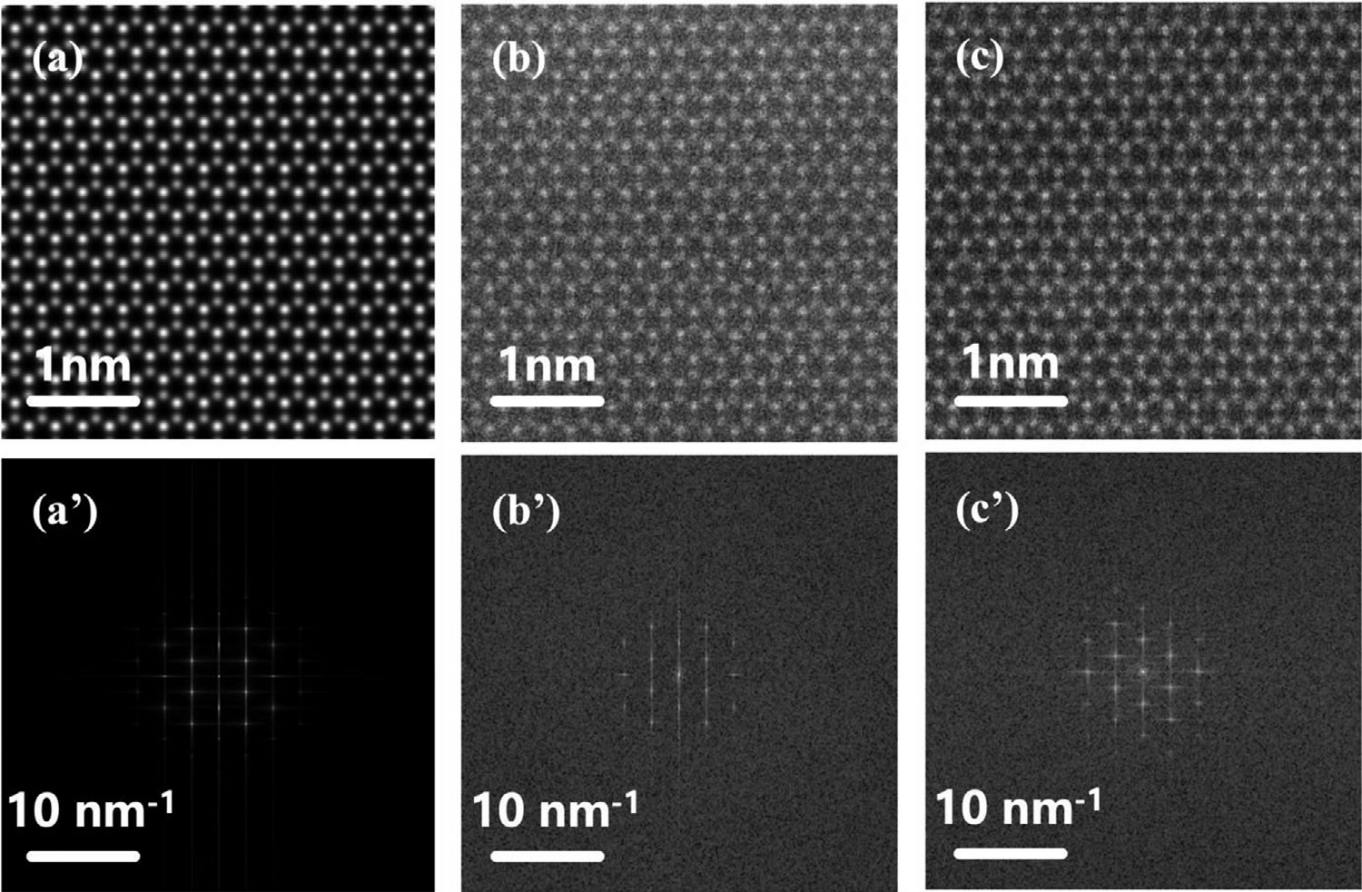
Representative images and their power spectra of raw simulated, STEMDiff‐generated images and experimental images of monolayer WSe_2_. The three columns of the figure are arranged in accordance with the aforementioned conditions, with the first row a–c) representing the real‐space STEM image for each case and the second row (a’‐c’) representing the corresponding power spectrum. The power spectra were obtained using the Fast Fourier Transform module from NumPy^[^ ^34^ ^]^ and visualized on a logarithmic scale. Scale bars are equal to 1 nm.

The STEMDiff approach fundamentally reconceptualizes this challenge by implementing a physics‐informed conditional diffusion process. Although trained on high‐fidelity STEM images derived from crystal structures, the model can, during inference, convert simple binary structural representations into STEM imagery that is indistinguishable from experimental results. **Figure** **2** presents a systematic comparison that reveals the capacity of STEMDiff to capture both spatial and spectral characteristics of experimental images. Particularly noteworthy is the accurate reproduction of high‐frequency spectral components visible in the Fourier transforms (Figure 2a'‐c'), which conventional simulations consistently fail to capture.

Beyond visual fidelity, STEMDiff demonstrates competitive computational efficiency by circumventing the sequential sampling iterations required by traditional Denoising Diffusion Implicit Models (DDIM).^[^ ^33^ ^]^ This optimization enables the generation of high‐resolution (256 × 256 pixel) experimentally authentic STEM images within milliseconds rather than seconds, representing a substantial computational acceleration that facilitates large‐scale dataset creation for ML applications.

While the current implementation specifically addresses WSe_2_ monolayer systems, the underlying framework possesses inherent extensibility to diverse crystalline structures and material compositions through appropriate expansion of the training dataset. This capability establishes a transformative pathway for automated STEM image generation that eliminates the requirement for laborious manual simulation parameter optimization, fundamentally advancing materials characterization workflows.

### 3.2 Quantitative Assessment of STEMDiff Image Authenticity

To objectively quantify the experimental authenticity of STEMDiff‐generated images beyond subjective visual assessment, we implemented a multi‐metric statistical evaluation framework comparing our approach with both conventional simulations and actual experimental acquisitions.

The primary quantitative benchmark employed in the analysis is the Fréchet Inception Distance (FID),^[^ ^35^ ^]^ a statistically robust metric that quantifies distributional similarity between generated and authentic image populations within a high‐dimensional feature space. Unlike simplistic pixel‐level comparisons, FID captures sophisticated perceptual similarities by leveraging the feature extraction capabilities of pretrained deep neural networks. The computational framework first processes both authentic experimental and generated images through an Inception‐v3 network,^[^ ^35^ ^]^ extracting high‐dimensional feature representations that encapsulate complex visual attributes. These feature distributions are then modeled as multivariate Gaussians, with the FID score quantifying their statistical divergence:

(7) $$\begin{array}{c} FID\;\left(E,G\right)=∥\mu_{E}-\mu_{G}{∥}^{2}+Tr\left(\Sigma_{E}+\Sigma_{G}-2{\left(\Sigma_{E}\Sigma_{G}\right)}^{0.5}\right) \end{array}$$

where µ_*E*_, and µ_*G*_ represent the mean of the features from experimental and generated images, respectively; Σ_*E*_ and, Σ_*G*_ are the covariance of the features of experimental and generated images, respectively. This formulation evaluates both positional disparities between feature clusters and the distinctive patterns of feature variability and interdependence. A lower FID score indicates greater similarity between generated and actual images.

**Figure 3:**
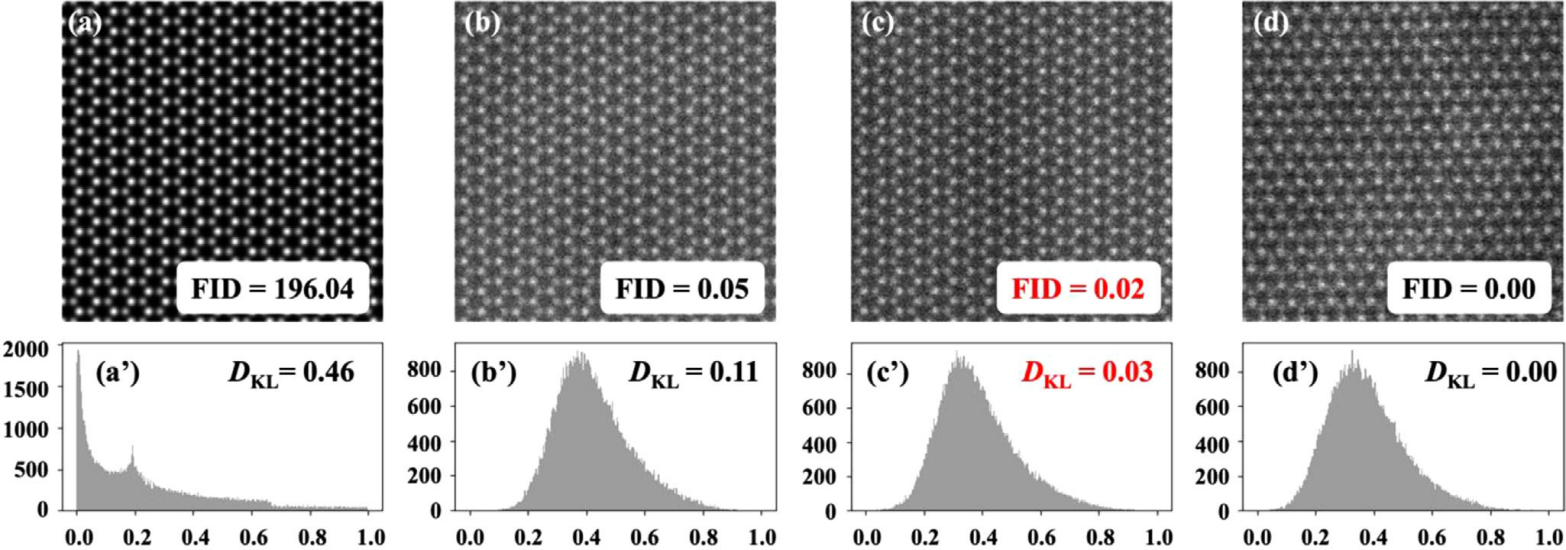
Quantitative measurement of the generated dataset using FID and KL divergence (*D*_KL_) for the WSe_2_ STEM image. a) noise‐free simulated images, b) simulated images with manually added noise, c) STEMDiff generated images, and d) Experimental STEM images. (a’‐d’) Histograms of normalized pixel intensity are calculated for each image dataset, with each histogram being normalized so that the probability distribution sums to unity. The *D*_KL_ of each data set with the experimental histogram is labeled as *D*_KL_ in the top right corner of each histogram, with the lowest non‐zero value marked in red.

Our comparative analysis (**Figure** **3**) evaluated three distinct image categories against experimental references: 1) non‐augmented computational simulations, 2) manually enhanced simulations with artificial noise profiles, and 3) STEMDiff‐generated images. The results demonstrate that the competitive performance of STEMDiff achieves an FID score of 0.02, reflecting a 17 fold improvement over previous state‐of‐the‐art GAN‐based methods reported by Khan et al.^[^ ^20^ ^]^ (FID = 0.35) and nearly four orders of magnitude superior to non‐augmented simulations (FID = 196.04).

To further validate these findings through complementary statistical measures, we calculated the KL divergence^[^ ^36^ ^]^ (*D*_KL_) between the pixel intensity distributions of each image category and experimental references. The *D*_KL_ provides an information‐theoretic quantification of distributional dissimilarity:

(8) $$D_{KL}\;\left(P∥Q\right)=\sum_{i}P\left(i\right)\;\log\frac{P\left(i\right)}{Q\left(i\right)}$$

where P represents the normalized pixel intensity distribution of experimental images, and Q corresponds to the distribution of generated images. A smaller *D*_KL_ value indicates similarity between distributions P and Q.

The pixel‐intensity histograms depicted in Figure 3a'–d' demonstrate that STEMDiff‐generated images exhibit statistical properties remarkably consistent with experimental acquisitions, with key distribution parameters (mean, variance, and higher‐order moments) closely aligned with authentic experimental data. This statistical congruence is quantitatively confirmed by the substantially lower *D*_KL_values for STEMDiff outputs compared to conventional simulation approaches.

These competitive statistical metrics directly result from the architectural elements of STEMDiff, particularly the NRBs, which enable the model to learn and reproduce the complex statistical distributions characteristic of experimental STEM observations. This capability extends beyond superficial visual similarity to encompass the nuanced noise characteristics, contrast variations, and high‐frequency spectral components that collectively define experimental authenticity.

### 3.3 Spectral Analysis of High‐Frequency Preservation Mechanisms

The notable experimental fidelity achieved by STEMDiff stems from its fundamental architectural innovations designed to overcome inherent limitations in conventional diffusion models. This section presents a detailed frequency‐domain analysis elucidating the mechanisms through which STEMDiff preserves critical high‐frequency components that define experimental STEM imagery.

Recent investigations into diffusion model behavior have revealed an intrinsic high‐frequency bias^[^ ^37^, ^38^ ^]^ that significantly impacts scientific image generation quality. Specifically, diffusion models exhibit a systematic tendency to prioritize reconstruction of low‐frequency image components while progressively attenuating high‐frequency details during the denoising process. This behavior reflects an underlying propensity of neural networks to preferentially learn low‐frequency representations during training, subsequently compromising the generation of sophisticated textural elements and fine structural details. For STEM imaging specifically, this limitation becomes particularly pronounced as experimental authenticity fundamentally depends on accurate reproduction of high‐frequency components encompassing atomic‐scale features, instrumental artifacts, and quantum noise patterns.

**Figure 4:**
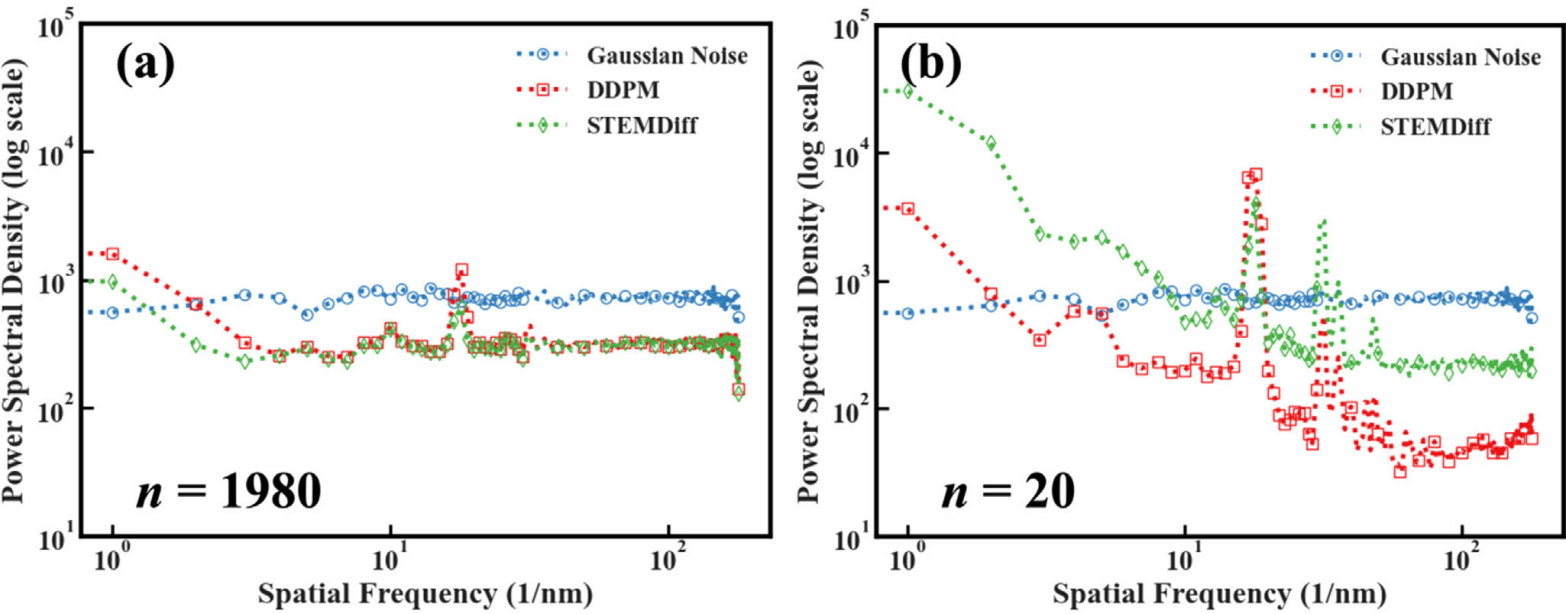
PSD analysis on the predicted ***ε*** of a vanilla U‐Net based DDPM, STEMDiff, and the ground truth (standard Gaussian noise). a) The power spectrum densities of the three at the beginning of the model's noise prediction (at n = 1980). b) The power spectrum densities of the three at the end of the model's noise prediction (at n = 20).

To systematically investigate this phenomenon, we conducted comparative spectral analyses between conventional diffusion models and our STEMDiff approach. **Figure** **4** presents Power Spectral Density (PSD) profiles of noise predictions generated by standard U‐Net‐based DDPM^[^ ^28^ ^]^ versus STEMDiff at critical timesteps in the denoising process (n = 20 and n = 1980). The results reveal a striking contrast: while conventional DDPM exhibits uniform spectral characteristics during early denoising stages (n = 1980), it demonstrates substantial attenuation of mid‐to‐high frequency components at terminal denoising stages (n = 20).

This phenomenon can be mechanistically understood through analysis of the denoising process dynamics. 1) During initial denoising iterations, the input signal predominantly contains high‐amplitude noise components, enabling the standard U‐Net architecture to effectively model the approximately uniform spectral characteristics of Gaussian noise through its skip‐connection mechanism. 2) As denoising progresses toward completion (n approaching 1), the residual noise components become progressively subtler and more structurally correlated with the underlying image content. 3) At this critical stage, the conventional U‐Net architecture demonstrates diminished capacity to capture high‐frequency noise modes, resulting in significant spectral distortion.

**Figure 5:**
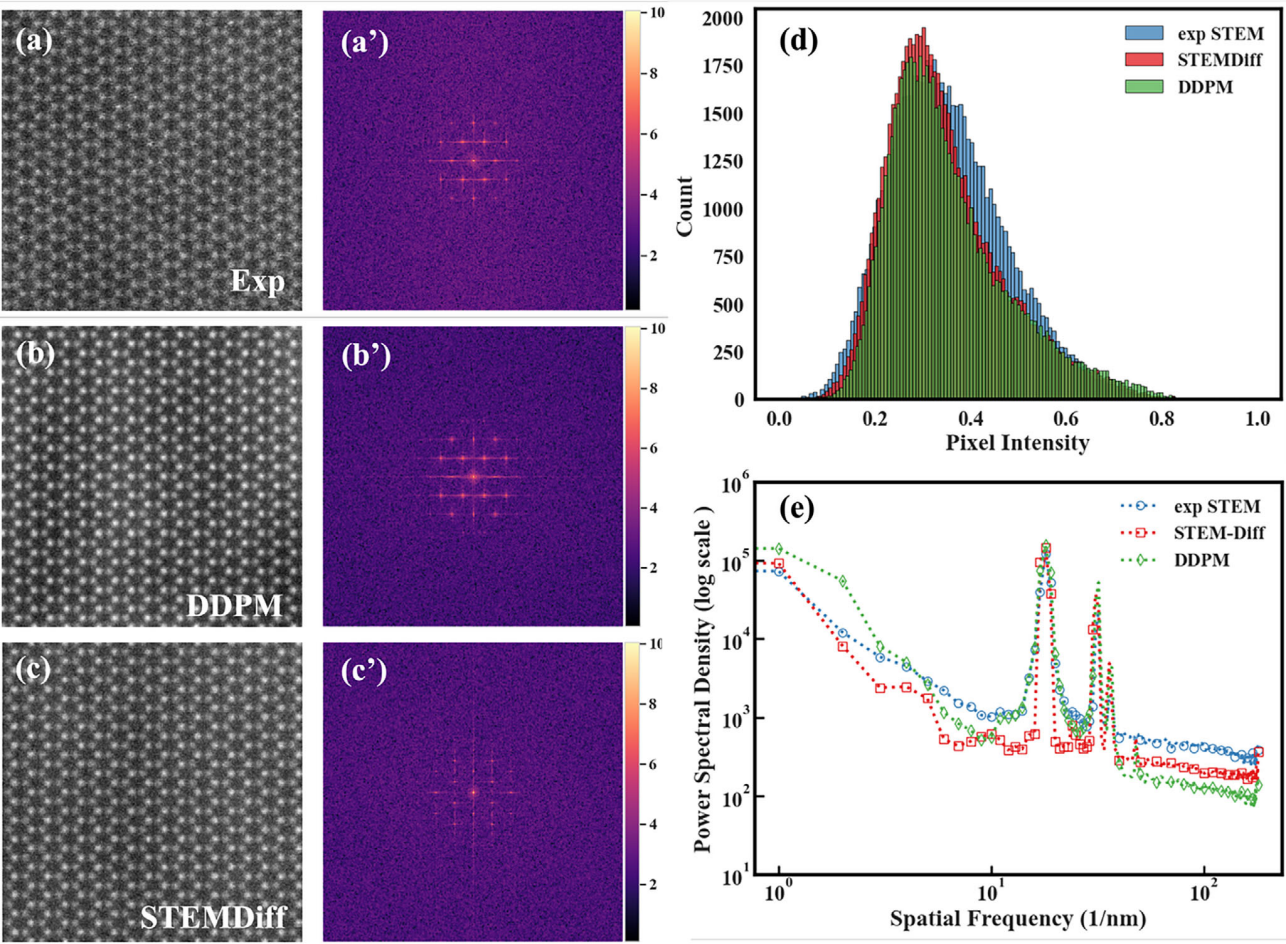
Disparity analysis between experimental STEM images, vanilla U‐Net‐based DDPM (DDPM), and STEMDiff‐generated STEM images. a–c) are STEM image and their corresponding Fourier Transformed version from a realistic experiment, DDPM and STEMDiff, respectively. From the Fourier space, it can be seen that the DDPM‐generated STEM image is lacking in high frequencies in comparison to the STEMDiff image. d) Statistical histograms of real‐space pixel values for all three, where the STEM image generated by STEMDiff has a mean and variance closer to the experimental STEM than the STEM image generated by DDPM. e) Comparison of the power spectral density of the experimental STEM image with that of the STEM image generated by DDPM and STEMDiff.

To further validate this analysis, we performed detailed comparative spectral investigations of experimental STEM images alongside those generated by conventional DDPM and STEMDiff (**Figure** **5**). While spatial‐domain representations (Figure 5a–c) exhibit subtle perceptual differences, Fourier analysis (Figure 5a'–c') reveals pronounced disparities in high‐frequency content between conventional DDPM outputs and experimental references, particularly evident in spectral regions beyond the primary diffraction lattice.

Quantitative PSD analysis (Figure 5e) confirms that STEMDiff preserves substantially higher spectral energy across mid‐to‐high frequency bands compared to conventional diffusion approaches. Furthermore, statistical analysis of intensity distributions (Figure 5d) demonstrates that STEMDiff outputs exhibit mean and variance parameters more closely aligned with experimental references than conventional DDPM generations.

The STEMDiff architecture directly addresses these limitations through the DWT‐based NRBs, which fundamentally transform the information flow dynamics during the denoising process. By selectively isolating and preserving high‐frequency components via wavelet decomposition, STEMDiff maintains critical spectral characteristics throughout the denoising trajectory. The spectral profiles in Figure 4 confirm that STEMDiff predictions at terminal denoising stages (n = 20) exhibit substantially improved preservation of high‐frequency components, with spectral characteristics more closely resembling the uniform distribution of authentic Gaussian noise.

This wavelet‐based approach leverages the inherent multi‐resolution capabilities of DWT to efficiently capture and preserve frequency information across multiple scales, providing a mathematically principled solution to the high‐frequency bias problem. Consequently, STEMDiff generates images that exhibit both visual and spectral congruence with experimental references, establishing a standard for experimental fidelity in computational STEM image generation.

### 3.4 Application of STEMDiff in Atomic Column Detection with Fully Convolutional Networks

Beyond theoretical performance metrics, the ultimate validation of the utility of STEMDiff lies in its capacity to support downstream analytical applications in materials characterization. To demonstrate this practical value, we implemented a fully convolutional network (FCN) for atomic column detection in experimental STEM imagery, trained exclusively on STEMDiff‐generated synthetic data.

Accurate localization of atomic columns represents a fundamental analytical challenge in materials characterization, with substantial implications for understanding structure‐property relationships. Traditional approaches for addressing this challenge broadly categorize into two methodological families: 1) real‐space techniques utilizing geometric fitting algorithms,^[^ ^39^, ^40^ ^]^ and 2) reciprocal‐space methods employing template matching.^[^ ^41^ ^]^ While geometrically intuitive, real‐space approaches typically require extensive manual parameter optimization and demonstrate limited robustness to experimental artifacts. Conversely, reciprocal‐space methods exhibit superior performance under idealized imaging conditions but suffer substantial degradation when confronted with beam‐induced specimen modifications,^[^ ^32^, ^42^ ^]^ contamination effects,^[^ ^18^, ^43^ ^]^ and other experimental variables.

Contemporary deep learning approaches offer compelling advantages for addressing these challenges through their capacity to learn complex image‐to‐structure mappings directly from data. However, their practical implementation has been severely constrained by the scarcity of appropriately annotated training datasets, as the acquisition and manual annotation of experimental STEM images represents a prohibitively resource‐intensive process.

Trained on STEM images produced via physical simulations, the STEMDiff framework supports an effective structure‐to‐image generative approach at inference time, effectively linking crystallographic representations with images that closely resemble real experimental results. To demonstrate this capability, we implemented an FCN with the architectural configuration to identify atomic positions of monolayer in experimental STEM images, comprising a symmetric encoder‐decoder structure with skip connections between corresponding resolution levels. This network was trained solely on synthetic data, and in this study, we take monolayer WSe2 and graphene structures as examples. The synthetic dataset was generated through a streamlined workflow incorporating: 1) programmatic extraction of atomic coordinates from crystallographic information files, 2) generation of binary structural maps with intensity peaks corresponding to atomic column positions, and 3) application of STEMDiff to transform these abstract representations into experimentally authentic STEM visualizations.

For the experimental implementation, we generated 400 binary structural images incorporating diverse geometric transformations (rotations, scaling, and translations) to ensure robust generalization. After processing through STEMDiff and subsequent quality filtering, 185 synthetic training examples were retained for FCN training. Despite its moderate size, the collection of training examples demonstrated remarkable efficacy in identifying atomic position coordinates within experimental STEM images.

**Figure 6:**
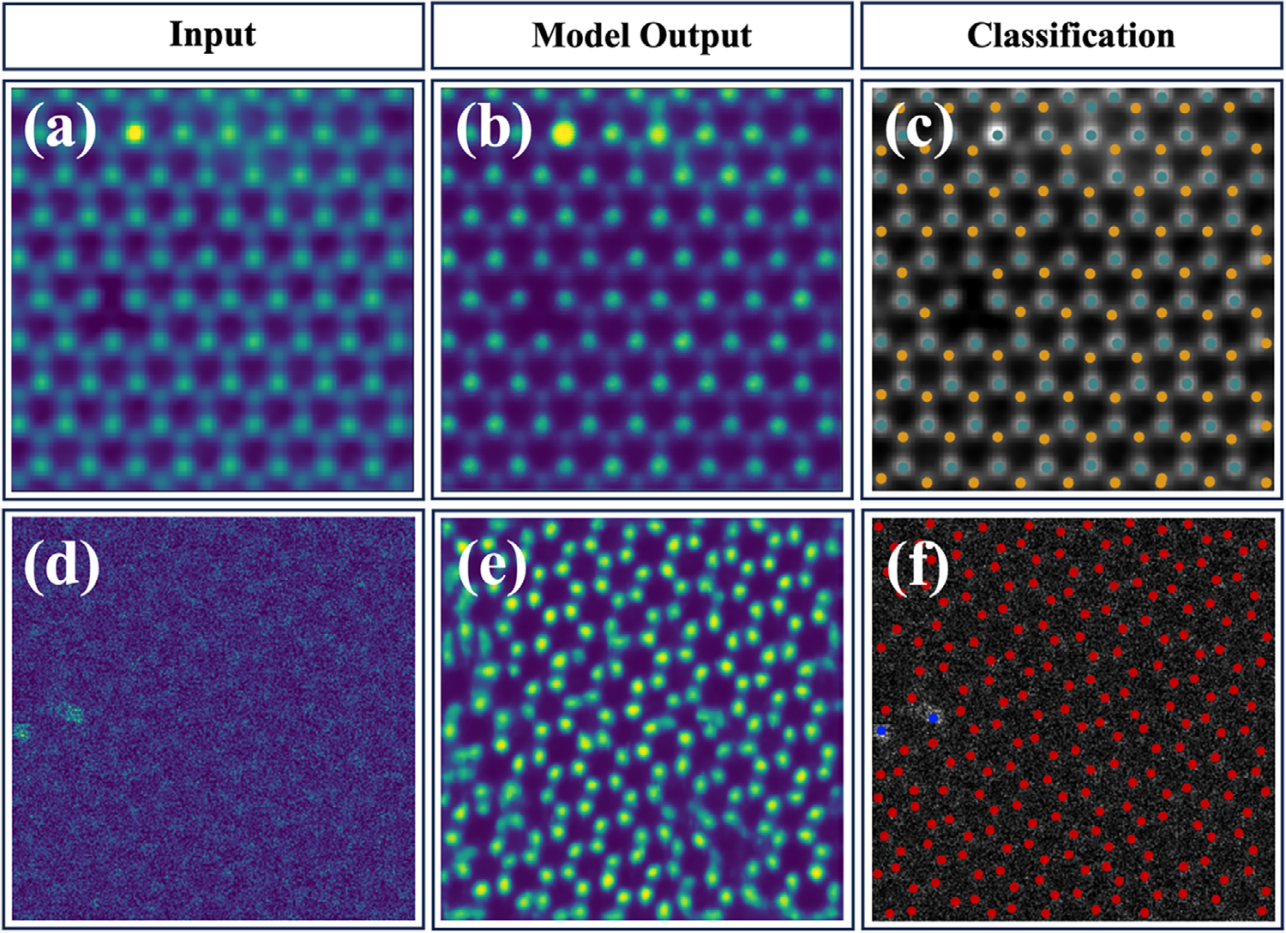
Atomic column detecting and elemental classification on experimental STEM images using an FCN trained exclusively on STEMDiff‐generated images. Subfigures are organized into three columns corresponding to distinct processing stages: Input(left), Model Output(middle), and Classification(right). a–c) present results of the WSe_2_ system: (a) the experimental STEM image of monolayer WSe_2_ (from Khan et al's study^[^ ^20^ ^]^), (b) the segmentation probability map produced by the trained FCN network, (c) the classification result showing identified atomic columns overlaid on the original STEM image, where yellow indicates Se and green indicates W. d–f) correspond to the graphene system: (d) the experimental STEM image of monolayer graphene (from Dyck's study^[^ ^44^ ^]^); (e) the FCN‐generated segmentation probability map, (f) the classification result with atomic positions annotated by predicted elemental species, where red denotes C atoms and blue denotes Si atoms.

To assess performance in realistic scenarios, we evaluated the FCN on two distinct experimental systems: monolayer WSe_2_ and monolayer graphene. Although trained solely on images generated by STEMDiff, the model successfully detected atomic column positions in both material systems without requiring fine‐tuning or domain adaptation. **Figure** **6** displays representative outcomes for each material under practical imaging conditions, such as low signal‐to‐noise ratio (SNR). Each row pertains to one material system and depicts the complete inference workflow, from the raw experimental input (left), to the segmentation probability map (middle), and finally to the elemental classification of atomic columns (right). For the WSe_2_ example (Figure 6a–c), yellow and green markers indicate predicted Se and W atoms, respectively. For the graphene example (Figure 6d–f), red and blue markers denote carbon atoms and substitutional Si atoms, respectively.

This practical demonstration establishes several significant advantages of the STEMDiff‐FCN workflow: 1) dramatic reduction in manual annotation requirements through automated label generation, 2) remarkable data efficiency, with high‐performance models trained on just ≈120 synthetic examples, 3) preservation of physically meaningful high‐frequency features critical for structural analysis, and 4) closed‐loop validation of experimental fidelity of STEMDiff through successful transfer learning to authentic experimental conditions.

While taking WSe_2_ and graphene monolayers as examples in this study, the methodology exhibits inherent extensibility to diverse material systems and experimental configurations, establishing a generalizable framework for automated structural analysis in electron microscopy. This capability represents a crucial bridge between theoretical simulation and experimental analysis, substantially accelerating materials characterization workflows while reducing human intervention requirements.

## 4 Conclusion

The development of STEMDiff represents a significant advancement in addressing a fundamental challenge in data‐driven materials characterization, i.e., the creation of realistic, physics‐informed training data for ML applications in electron microscopy. By combining conditional diffusion models with Discrete Wavelet Transform‐based Noise Retaining Blocks, we've successfully overcome the high‐frequency bias inherent in traditional generative networks, enabling the preservation of critical atomic‐scale details and experimental artifacts that define authentic STEM imagery. The competitively low FID scores (0.02) and KL divergence metrics demonstrate that STEMDiff generates images nearly indistinguishable from experimental data, outperforming conventional approaches by orders of magnitude. More importantly, the practical utility of this approach is validated through downstream atomic column detection tasks, where FCNs trained solely on synthetic STEMDiff data achieve remarkable precision on real experimental images containing complex noise patterns and contamination artifacts. This bridging of the simulation‐experiment gap opens new avenues for accelerated materials discovery by enabling rapid, autonomous analysis of electron microscopy data with minimal human intervention. The methodology offers a versatile framework applicable to diverse material systems and imaging modalities. STEMDiff thus offers a novel path for physics‐informed synthetic data generation that could fundamentally transform how one trains ML models for materials characterization and discovery, with extensions to fields like environmental science.

## Conflict of Interest

The authors declare no conflict of interest.

## Supporting information



Supporting Information

## Data Availability

The data that support the findings of this study are available from the corresponding author upon reasonable request.
