# Supplementary material for: STEMDiff: A Wavelet‐Enhanced Diffusion Model for Physics‐Informed STEM Image Generation
Source: Adv Sci (Weinh). 2025 Sep 27;12(41):e08266. doi: 10.1002/advs.202508266 (PMC12591186; doi:10.1002/advs.202508266)
Supplement: Supplementary file 1 — Supporting Information [file ADVS-12-e08266-s001.docx]

**Supporting Information for**

**STEMDiff : A Wavelet-Enhanced Diffusion Model for Physics-Informed STEM Image Generation**

*Yihui Bao* ^a^*, Xinyi Lu* ^a^*, Yanyan Xia* ^a^*, Zhencheng Ye*^, a^, Houyang Chen*^, b, c^*

Yihui Bao, Xinyi Lu, Yanyan Xia, Prof. Dr. Zhencheng Ye

^a^ School of Information Science and Engineering,

East China University of Science and Technology,

Shanghai 200237, P. R. China.

E-mail: yzc@ecust.edu.cn (Z. Ye)

Prof. Dr. Houyang Chen

^b^ Chongqing Institute of Green and Intelligent Technology,

Chinese Academy of Sciences,

Chongqing 400714, P. R. China.

^c^ Chongqing School,

University of Chinese Academy of Sciences,

Chongqing 400714, P. R. China.

E-mail: chenhouyang@cigit.ac.cn (H. Chen)

**Section S1. Implementation and Training Details**

**Hardware and Environment Setup**: All experiments were conducted on a workstation equipped with an NVIDIA GeForce RTX 4090 D GPU. The model was implemented using Python 3.8.10, CUDA 11.8, and PyTorch 2.0.0.

**Network Architecture**: The denoising model is based on a modified U-Net backbone (adapted from SR3) in which all skip connections are replaced with our proposed Noise Retaining Blocks (NRBs). The model takes as input a concatenation of Gaussian noise and a binary label map (grayscale image), resulting in 2-channel input images.

**Hyperparameters**: During training, we use 256×256 resolution images, a batch size of 16, and a total of 100,000 iterations. The initial learning rate is set to 1e-4 and decays by a factor of 0.7 every 0.4 million iterations. We employ 2000 forward diffusion steps during training, and 20 reverse steps during testing via DDIM sampling.

**Noise Schedule**: Our noise schedule follows a standard linear variance scaling across 2000 steps as used in DDPM, ensuring a gradual transformation of input images into near-pure Gaussian noise. This is implemented analytically as described in Section 2.1a of the main manuscript.

**Data Augmentation & Realism Enhancement**: In addition to the simulated raw images generated via **abTEM**, we apply an enhancement pipeline that introduces realistic noise components — including Poisson noise, additive Gaussian noise, scan-line jitter, geometric distortions (shear, compression), and contamination background injection. Details of this augmentation strategy are documented in Section 2.2 of the main manuscript.

**Regularization and Stability**: To mitigate divergence issues, especially from high-frequency amplification during early training, we incorporate dropout layers throughout the encoder-decoder network architecture, including in the NRBs. Additionally, our proposed DWT-based NRBs structurally enforce preservation of localized high-frequency components while avoiding instability from overfitting noise. This design has shown improved stability compared to vanilla U-Net-based diffusion models, as evidenced in our spectral analysis (see Section 3.3, *Figure 4* & *Figure 5*).

**Section S2. More detailed information for Figure 3**

Figure 3a (referred to as “Raw simulated images”) was generated using abTEM by importing atomic structures, setting the electrostatic potential and probe wave function, defining the scan region and detector geometry, and integrating the measurements. A pixel interpolation (sampling rate: 0.05 Å) and Gaussian filtering (σ = 0.3) were subsequently applied to simulate partial spatial coherence. This image does not include Poisson noise, thermal scattering, or realistic lens aberrations.

Figure 3b (referred to as “Simulated images with manually added noise”) was generated by applying abTEM’s poisson_noise interface on the measurement object to simulate different electron doses per unit area. This effectively accounts for stochastic effects such as thermal scattering and lens aberrations. In addition, we simulated experimental noise characteristics, such as probe scattering, sample drift, and background contamination, using custom Python functions. This subfigure reflects the kind of physically realistic simulation the reviewer recommended.
